# Liver Transplantation in Severe Hemophilia A: A Two-Patient Case Series with Divergent Long-Term Outcomes

**DOI:** 10.3390/reports9040342

**Published:** 2026-10-10

**Authors:** Alexey Vasilyevich Shabunin, Pavel Alexeevich Drozdov, Oksana Nikolaevna Levina, Sergey Andreevich Astapovich, Daria Andreevna Teplyuk, Dzhennet Agamovna Beibalaeva

**Affiliations:** 1 Botkin Hospital, Moscow Healthcare Department, 5, 2nd Botkinsky Proezd, Moscow 125284, Russia; glavbotkin@zdrav.mos.ru (A.V.S.); dc.drozdov@gmail.com (P.A.D.); levinaoks@mail.ru (O.N.L.); teplyuk_d_a@staff.sechenov.ru (D.A.T.); dzhengadzh@icloud.com (D.A.B.); 2 Russian Medical Academy of Continuous Professional Education, Ministry of Health of the Russian Federation, 2/1 Barrikadnaya Street, Moscow 125993, Russia

**Keywords:** factor VIII, hemophilia A, hepatocellular carcinoma, hepatitis C virus, liver transplantation, thromboelastography, long-term outcome

## Abstract

**Background and Clinical Significance:** Before effective viral inactivation of plasma-derived factor concentrates, many patients with hemophilia acquired hepatitis C virus infection and later developed cirrhosis or hepatocellular carcinoma. Liver transplantation treats end-stage liver disease and may also restore factor VIII production; however, long-term oncologic outcomes in this setting are rarely reported. **Case Presentation:** Two men with severe hemophilia A (baseline factor VIII activity < 1%) and hepatitis C-related cirrhosis underwent deceased-donor orthotopic liver transplantation. Both received an extended-half-life recombinant factor VIII (efmoroctocog alfa; 33,000 and 35,000 IU in total) tapered until postoperative days 13–14, and intraoperative hemostasis was guided by conventional coagulation tests and thromboelastography. Neither patient had major bleeding or thrombotic events, and replacement therapy was not required thereafter. In Patient 1, graft function remained satisfactory 45 months after transplantation, with factor VIII activity of 62.5%. Patient 2 was transplanted for hepatocellular carcinoma outside the Milan criteria (two lesions of 6.8 and 2.7 cm; alpha-fetoprotein 110–150 ng/mL) under center-specific expanded criteria, without bridging therapy. Factor VIII activity remained clinically sufficient, but pulmonary nodules suspected 20 months after transplantation progressed; atypical pulmonary resections 45 months after transplantation confirmed metastatic hepatocellular carcinoma, and lenvatinib was initiated. **Conclusions:** Liver transplantation can provide durable correction of severe hemophilia A without long-term replacement therapy. However, correction of the coagulation disorder does not eliminate risks related to the original liver disease; in patients transplanted for hepatocellular carcinoma, particularly beyond conventional criteria, long-term outcome remains dependent on tumor burden, tumor biology, candidate selection, and sustained oncologic surveillance.

## 1. Introduction and Clinical Significance

Hemophilia A is an X-linked inherited bleeding disorder caused by deficient factor VIII activity. Before donor screening and effective viral inactivation became routine, repeated exposure to pooled plasma-derived coagulation-factor concentrates placed many patients at high risk of hepatitis C virus infection. Cirrhosis, portal hypertension, liver failure, and hepatocellular carcinoma consequently became important late complications in aging populations with hemophilia [1].

Liver transplantation is indicated for the same accepted hepatic conditions in patients with hemophilia as in other transplant candidates. In addition to replacing the diseased liver, transplantation can restore clinically sufficient factor VIII activity and eliminate the need for long-term replacement therapy. Early reports and later series demonstrated rapid and sustained correction of the bleeding phenotype after successful transplantation [2,3,4,5,6,7]. Perioperative management nevertheless remains challenging because severe baseline factor deficiency, portal hypertension, thrombocytopenia, surgical blood loss, and rapidly changing endogenous factor production after graft reperfusion must all be considered [6,8].

Liver transplantation in hemophilia A is therefore not a new procedure. It has been described in numerous case reports and institutional and multicenter series, including living-donor transplantation, transplantation in patients with factor VIII inhibitors, and transplantation for hepatitis C-related hepatocellular carcinoma [9,10,11,12,13]. The early perioperative course of the two patients described here has been reported previously in Russian [14]. Most published reports have emphasized perioperative hemostasis or short-term factor correction. This case series extends follow-up to 45–50 months, provides detailed perioperative factor VIII and hemostatic data, and describes two patients with similar hematologic outcomes but divergent long-term clinical courses, including histologically confirmed metastatic recurrence of hepatocellular carcinoma. The report was prepared in accordance with the CARE guidelines [15].

## 2. Case Presentation

### 2.1. Patient 1

A man in his late 40s had been diagnosed with severe hemophilia A in childhood, with baseline factor VIII activity below 1%, and had received factor VIII replacement throughout his life. Antibodies to hepatitis C virus were first detected in 1991. Hepatitis C-related cirrhosis was diagnosed in 2018. Treatment with sofosbuvir and daclatasvir in 2020 resulted in a sustained virologic response.

In 2021, the patient developed progressive edema and ascites, recurrent epistaxis, iron-deficiency anemia, leukopenia, thrombocytopenia, hypoalbuminemia, and mild hyperbilirubinemia. Upper gastrointestinal endoscopy demonstrated grade II esophageal varices. Contrast-enhanced computed tomography showed cirrhosis, extensive portosystemic collateralization, splenomegaly, and ascites. At listing, the Model for End-Stage Liver Disease score was 12 and the Child-Pugh score was 8 (class B).

**Figure 1:**
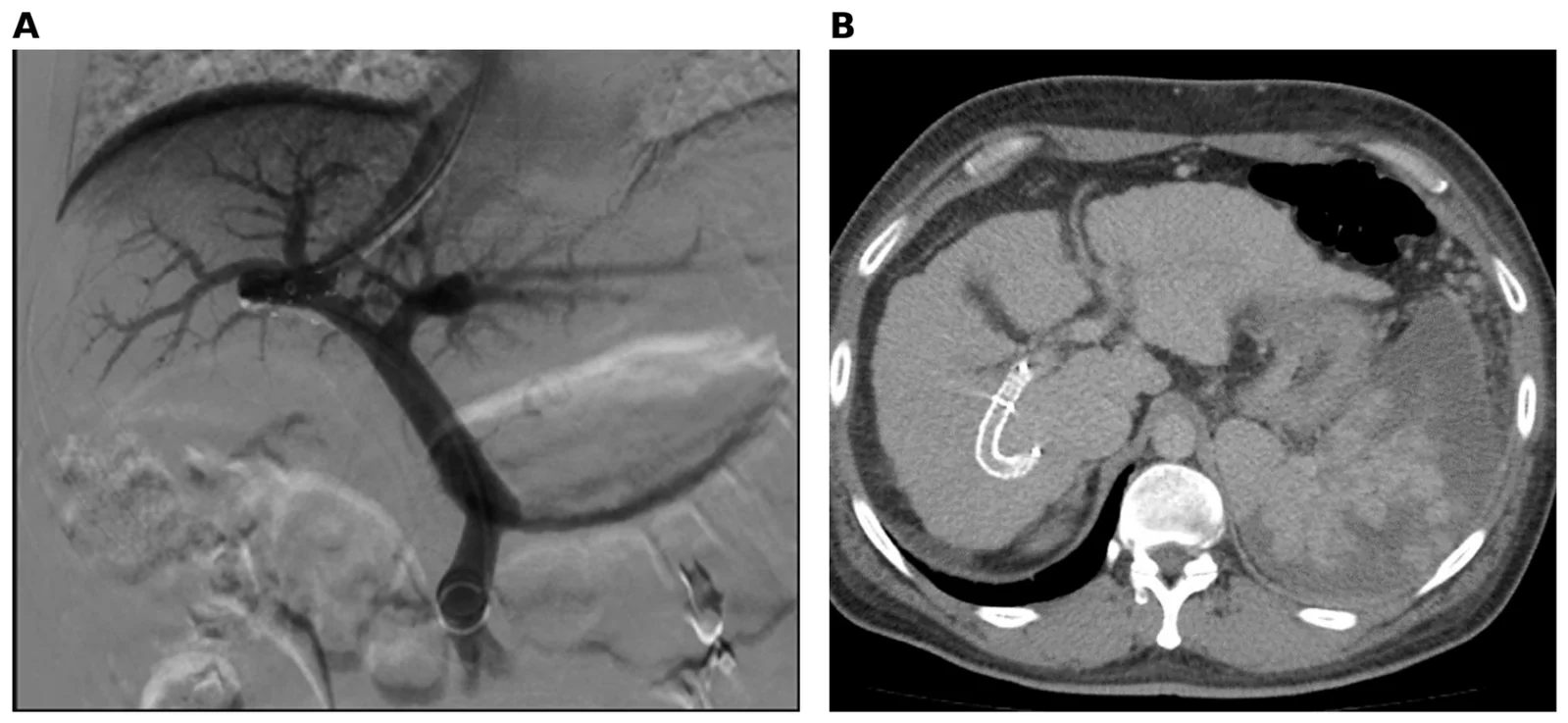
(**A**) Portography during transjugular intrahepatic portosystemic shunt placement, demonstrating the intrahepatic communication between the portal venous system and the right hepatic vein. (**B**) Follow-up contrast-enhanced computed tomography showing the shunt in the expected position and post-embolization ischemic changes in the spleen. The images are original clinical images owned by the authors.

Because of clinically significant portal hypertension and marked thrombocytopenia, transjugular intrahepatic portosystemic shunt placement and endovascular splenic artery embolization were performed in September 2021. The platelet count increased from 45 × 10^9^/L to 101 × 10^9^/L after two weeks. Follow-up imaging demonstrated a patent shunt and post-embolization ischemic changes in the spleen (Figure 1).

Deceased-donor orthotopic liver transplantation was performed in April 2022. Perioperative factor VIII replacement and intraoperative hemostatic management are described in Section 2.3. No unplanned rescue doses were required. The postoperative course was uncomplicated, with no clinically significant bleeding or thrombosis. Maintenance immunosuppression consisted of prolonged-release tacrolimus.

Factor VIII replacement was discontinued after postoperative day 13. At the latest available assessment in January 2026, graft function remained satisfactory: alanine aminotransferase 14 U/L, aspartate aminotransferase 16 U/L, total bilirubin 13 μmol/L, albumin 44 g/L, and platelet count 238 × 10^9^/L. Factor VIII activity was 62.5% without replacement therapy. No rejection or other clinically significant late extrahepatic or immune-mediated complications were documented. The patient remained clinically stable 45 months after transplantation.

### 2.2. Patient 2

A man in his early 50s had severe hemophilia A with baseline factor VIII activity below 1% and had received factor VIII replacement since childhood. Hepatitis C-related cirrhosis was diagnosed in 2008. Several interferon-based antiviral courses were administered, and a sustained virologic response was documented after the final course in 2014.

The patient had recurrent esophagogastric variceal bleeding, surgical oversewing of gastric varices in 2015, and repeated endoscopic variceal ligation in 2016 and 2017. Computed tomography in July 2021 revealed a segment IVa liver lesion measuring 50 × 45 × 34 mm. By December 2021, the lesion had increased to 68 × 50 × 43 mm, and a second segment IVb lesion measuring 27 × 17 mm was identified. The radiologic category was reported as Liver Imaging Reporting and Data System (LI-RADS) 4, and serial serum alpha-fetoprotein (AFP) values increased from 110 to 130 and 150 ng/mL. At listing, the Model for End-Stage Liver Disease score was 14 and the Child-Pugh score was 7 (class B).

With two lesions, the largest measuring 6.8 cm, the patient was outside the Milan criteria [16] and also outside the University of California San Francisco (UCSF) and up-to-seven criteria [17,18]. The center-specific expanded oncologic criteria allowed consideration for liver transplantation in patients with a pretransplant AFP level below 400 ng/mL and a total tumor diameter below 10 cm; the total tumor diameter in Patient 2 was 9.5 cm, and all AFP values remained below the institutional threshold. According to the Metroticket 2.0 model, the estimated 5-year hepatocellular carcinoma (HCC)-specific survival was approximately 54–58%, below the 70% benchmark proposed for that model [19]. Following multidisciplinary assessment, this estimate was considered acceptable under the center-specific criteria, and the patient was accepted for transplantation. Neither bridging nor downstaging locoregional therapy was performed before transplantation; the specific rationale was not documented in the available retrospective records.

Deceased-donor orthotopic liver transplantation was performed in February 2022. Perioperative management is described in Section 2.3. No unplanned rescue doses were required, and no major bleeding or thrombotic events occurred. The available explant pathology report described moderately differentiated hepatocellular carcinoma with negative margins, no vascular invasion, and no perineural invasion; the recorded pathologic stage was pT1 Nx Mx. The report did not specify the number of viable tumor nodules or the maximal diameter of viable tumor, and the original slides and complete histologic mapping were not available for review. Maintenance immunosuppression consisted of prolonged-release tacrolimus and everolimus.

Factor VIII replacement was discontinued after postoperative day 14. Factor VIII activity was 59.3% in March 2023 and 44.5% in April 2024 without replacement therapy. Liver graft function remained satisfactory, and cross-sectional oncologic surveillance was performed approximately every six months.

In October 2023, small bilateral pulmonary nodules were first considered suspicious for metastatic disease; however, cytologic examination of bronchoalveolar lavage fluid did not show malignant cells. By April 2025, the nodules had enlarged, including a right upper-lobe lesion measuring 22 × 14 mm, and additional abdominal lesions were reported. Positron-emission tomography in June 2025 demonstrated low fluorodeoxyglucose uptake in the pulmonary lesions.

Because of persistent radiologic suspicion and interval progression, atypical resections of right pulmonary lesions were performed in November 2025. Histologic and immunohistochemical findings confirmed metastatic hepatocellular carcinoma. Systemic therapy with lenvatinib was initiated in January 2026. Computed tomography in April 2026 showed enlargement of a left lower-lobe metastatic nodule to 9 mm and progression of multiple peritoneal lesions. Histologic confirmation was delayed because the initial bronchoalveolar lavage cytology was negative and the pulmonary lesions demonstrated low fluorodeoxyglucose uptake; tissue diagnosis was ultimately obtained after continued radiologic progression. Thus, durable correction of hemophilia and preserved graft function coexisted with progressive recurrent hepatocellular carcinoma. The clinical characteristics and outcomes of both patients are summarized in Table 1. The timeline of recurrence in Patient 2 is presented in Table 2.

### 2.3. Perioperative Factor VIII Replacement and Hemostatic Management

Perioperative replacement therapy in both patients was performed with efmoroctocog alfa (Elocta), an extended-half-life recombinant factor VIII Fc fusion protein, with a target perioperative factor VIII activity of approximately 100 IU/dL. Patient 1 received 4000 IU (51.3 IU/kg) 45 min before skin incision and an additional 2000 IU (25.6 IU/kg) approximately 1 h after graft reperfusion. Patient 2 received 4000 IU (55.6 IU/kg) 30 min before skin incision and an additional 2000 IU (27.8 IU/kg) approximately 45 min after reperfusion. From postoperative days 1 to 3, both patients received 2000 IU every 12 h. Patient 1 subsequently received 2000 IU once daily on postoperative days 4–6, 1500 IU on days 7–10, and 1000 IU on days 11–13. Patient 2 received 2000 IU once daily on postoperative days 4–7, 1500 IU on days 8–11, and 1000 IU on days 12–14. The total administered doses were 33,000 IU and 35,000 IU, respectively (Table 3). No rescue doses were required.

Neither patient had a known history of factor VIII inhibitors; however, the results of formal pretransplant inhibitor assays were not available in the retrospective records. Factor VIII activity was included in intraoperative laboratory monitoring, but a complete series of perioperative factor VIII values could not be retrieved.

Intraoperative coagulation management was guided by serial conventional coagulation tests and thromboelastography (TEG). Hemoglobin concentration, platelet count, prothrombin time, international normalized ratio, activated partial thromboplastin time, fibrinogen concentration, and factor VIII activity were assessed before surgery, during the anhepatic phase, after graft reperfusion, and at the end of the procedure. TEG was performed at baseline, during the anhepatic phase, and after reperfusion. Blood components were administered according to clinical assessment and laboratory and TEG findings. Patient 1 received four units of fresh-frozen plasma, one apheresis platelet unit, and two units of packed red blood cells. Patient 2 received three units of fresh-frozen plasma and two units of packed red blood cells; platelet transfusion was not required. Neither patient received fibrinogen concentrate, cryoprecipitate, tranexamic acid, prothrombin complex concentrate, or recombinant activated factor VII.

## 3. Discussion

These cases demonstrate sustained, clinically sufficient factor VIII activity after liver transplantation in patients with severe hemophilia A. Both patients discontinued replacement therapy after transplantation and had no clinically important bleeding event during the available follow-up. This outcome is consistent with the earliest clinical reports and subsequent institutional and multicenter series, in which factor activity increased rapidly after graft reperfusion and remained sufficient without long-term replacement therapy [2,3,4,5,6,7]. Contemporary cohort data also support durable factor correction many years after transplantation; in a multicenter cohort, median factor VIII activity was 0.63 IU/mL at a median of 9.2 years after transplantation [20]. The decline of factor VIII activity to 44.5% in Patient 2 remains clinically sufficient but supports periodic monitoring.

Perioperative hemostatic management remains central to a successful outcome. Major surgery in severe hemophilia generally requires factor VIII activity near the normal range during the operation and early postoperative period, followed by progressively lower targets during wound healing [8,21]. After graft reperfusion, endogenous factor VIII production may increase rapidly; therefore, replacement therapy should be adjusted using frequent laboratory assessment rather than a fixed postoperative schedule alone. In published series, factor support was often no longer required within 72 h after transplantation [4], and in individual reports replacement was discontinued on postoperative day 3 or within the first postoperative day once measured factor VIII activity exceeded 80% [11,12]. In the present cases, replacement with an extended-half-life product was tapered until postoperative days 13–14, no rescue dosing was required, and no major hemorrhagic or thrombotic complication occurred. This fixed taper was conservative, and because complete serial factor VIII measurements were unavailable, we cannot determine whether earlier discontinuation would have been possible. Pretransplant inhibitor testing is also important: recipient inhibitors require specific eradication and bypassing strategies [12], donor-derived inhibitors can cause acquired hemophilia after transplantation [22], and de novo high-titer inhibitors after transplantation have been associated with life-threatening complications [23].

Intraoperative coagulation management during liver transplantation has changed substantially over the past two decades. Transfusion requirements have declined, transfusion-free transplantation has become more frequent, and conventional coagulation tests are recognized as poor guides to the rebalanced hemostasis of cirrhosis, in which bleeding and thrombotic risks coexist [24]. Viscoelastic tests, including TEG and rotational thromboelastometry (ROTEM), assess clot initiation, strength, and fibrinolysis in whole blood at the point of care and allow goal-directed administration of plasma, platelets, fibrinogen, and antifibrinolytic agents. ROTEM-based algorithms have been evaluated in before-and-after studies [25], and a randomized trial showed a reduction in total blood product transfusion, particularly of fresh-frozen plasma [26]. A 2023 meta-analysis of 17 studies including 5345 patients found reduced red blood cell and plasma transfusion but greater cryoprecipitate use with viscoelastic guidance, without effects on mortality or length of stay; the certainty of evidence was moderate to very low [27]. Expert panel recommendations favor viscoelastic-guided management in liver transplantation [28], whereas the broader evidence base in bleeding patients remains limited [29]. No viscoelastic algorithm specific to hemophilia has been validated, and factor VIII activity must still be measured directly. In our patients, TEG was combined with conventional tests and factor VIII activity to guide component therapy, and transfusion requirements were modest.

Patient 1 also illustrates individualized pretransplant treatment of severe portal hypertension and hypersplenism. Transjugular intrahepatic portosystemic shunt placement combined with splenic artery embolization was followed by a substantial increase in platelet count. This observation should not be interpreted as evidence supporting routine use of this combination in patients with hemophilia because both procedures have independent risks and the present report cannot establish causality or comparative effectiveness.

Patient 2 provides the principal long-term lesson. Correction of the bleeding disorder did not alter the biological behavior of hepatocellular carcinoma. Liver transplantation for hepatitis C-related hepatocellular carcinoma in patients with hemophilia A has previously been reported, but most publications have focused on perioperative management rather than late oncologic outcomes [12,13]. Patient 2 was transplanted outside the Milan, UCSF, and up-to-seven criteria, and his estimated HCC-specific survival was below the Metroticket 2.0 benchmark; AFP-based models similarly show that tumor burden and AFP jointly determine recurrence risk [19,30]. Successful downstaging has been associated with favorable post-transplant outcomes in selected patients beyond the Milan criteria [31], but no locoregional therapy was performed in our patient. The lungs are the most frequent site of recurrence after transplantation for hepatocellular carcinoma, and pulmonary metastasectomy may provide prolonged survival in selected patients [32,33]. In our patient, small pulmonary lesions were suspected approximately 20 months after transplantation, but recurrence was not histologically confirmed until November 2025, after interval progression. Negative bronchoalveolar lavage cytology and low fluorodeoxyglucose uptake contributed to this delay; however, fluorodeoxyglucose avidity of hepatocellular carcinoma is heterogeneous and associated with tumor aggressiveness, so low uptake does not exclude metastatic disease [34]. The distinction between radiologic suspicion and pathologic confirmation is clinically important. Lenvatinib has shown efficacy and toxicity after liver transplantation comparable to those reported in non-transplant patients [35]. Previous series have identified pre-existing hepatocellular carcinoma and recurrent malignancy as important determinants of late outcome after liver transplantation in patients with hereditary bleeding disorders [5,6,7].

The available explant report recorded pT1 disease despite pretransplant imaging showing two lesions, the larger measuring 6.8 cm. In the American Joint Committee on Cancer staging system, pT1 denotes a solitary tumor without vascular invasion [36], which is inconsistent with the pretransplant imaging. The report did not specify the number of viable tumor nodules or the maximal viable tumor diameter, and complete histologic mapping and the original pathology slides were not available for this retrospective report, so the discrepancy could not be resolved. The pathologic stage should therefore be interpreted with caution and should not be taken as evidence of a low tumor burden. Radiologic–pathologic discordance is common after transplantation for hepatocellular carcinoma; in a registry analysis of 6842 recipients, one-third of patients within the Milan criteria on imaging exceeded them on explant pathology, and discordance was associated with recurrence and mortality [37]. Discordance in the opposite direction is also possible, because a proportion of LI-RADS 4 observations do not prove to be hepatocellular carcinoma [38].

Long-term outcomes after liver transplantation are determined not only by graft function and recurrence of the original liver disease. Late morbidity may also result from extrahepatic complications associated with chronic immunosuppression and long-term post-transplant care, including renal dysfunction, cardiovascular and metabolic disorders, opportunistic infections, de novo malignancies, and complications involving other organ systems. Immune-mediated complications, including acute or chronic rejection and autoimmune or inflammatory disorders such as IgA vasculitis (Henoch–Schönlein purpura), may also occur despite satisfactory graft function [39]. In recipients with hemophilia, de novo factor VIII inhibitors represent an additional disease-specific immune-mediated risk [23]. These potential complications require continued multidisciplinary surveillance. In the present cases, graft function remained satisfactory, and apart from recurrent hepatocellular carcinoma with extrahepatic metastatic progression in Patient 2, no additional clinically significant late extrahepatic or immune-mediated complications were documented during the available follow-up.

This report has limitations, including the two-patient sample, retrospective reconstruction of perioperative data, incomplete serial factor VIII measurements, unavailable formal pretransplant inhibitor assay results, absence of standardized pharmacokinetic monitoring, the undocumented rationale for omitting locoregional therapy in Patient 2, and lack of a comparison group. The report therefore cannot establish the superiority of a particular replacement regimen or pretransplant intervention. Its clinical value lies in demonstrating that successful hematologic correction and satisfactory graft function do not independently determine long-term survival.

## 4. Conclusions

With coordinated multidisciplinary care, deceased-donor liver transplantation can be performed without major hemorrhagic complications in selected patients with severe hemophilia A and can provide durable factor VIII correction without continued replacement therapy. Perioperative replacement should be individualized and guided by measured factor VIII activity and pretransplant inhibitor testing. Long-term prognosis, however, remains governed by the original indication for transplantation. For patients with hepatocellular carcinoma, particularly those transplanted beyond conventional criteria, meticulous candidate selection and prolonged oncologic surveillance remain essential despite normalization of hemostasis and stable graft function.

## Figures and Tables

**Table 1:** Comparative clinical characteristics and outcomes.

Characteristic	Patient 1	Patient 2
Hemophilia	Severe hemophilia A; baseline FVIII < 1%	Severe hemophilia A; baseline FVIII < 1%
Primary liver disease	HCV-related decompensated cirrhosis	HCV-related cirrhosis with HCC
Disease severity at listing	MELD 12; Child-Pugh B, 8 points	MELD 14; Child-Pugh B, 7 points
Major pretransplant features	Ascites, portal hypertension, esophageal varices, hypersplenism; TIPS and splenic artery embolization	Recurrent variceal bleeding; two liver lesions (68 and 27 mm), LI-RADS 4
Oncologic selection	Not applicable	AFP 110–150 ng/mL; outside Milan, UCSF, and up-to-seven criteria; within center-specific expanded criteria; no bridging or downstaging
Transplantation	Deceased-donor OLT, April 2022	Deceased-donor OLT, February 2022
Perioperative FVIII replacement	Efmoroctocog alfa, 33,000 IU in total; tapered through postoperative day 13	Efmoroctocog alfa, 35,000 IU in total; tapered through postoperative day 14
Major early bleeding or thrombosis	None	None
Explant pathology	Not applicable	Moderately differentiated HCC; negative margins; no vascular invasion; pT1 Nx Mx; number and diameter of viable tumors not reported
Post-transplant FVIII activity	62.5% in January 2026	59.3% in March 2023; 44.5% in April 2024
Long-term FVIII replacement	Not required	Not required
Long-term outcome	Stable graft function at 45 months	Pulmonary nodules suspected in October 2023; metastatic HCC confirmed in November 2025; lenvatinib initiated in January 2026

Abbreviations: AFP, alpha-fetoprotein; FVIII, factor VIII; HCC, hepatocellular carcinoma; HCV, hepatitis C virus; LI-RADS, Liver Imaging Reporting and Data System; MELD, Model for End-Stage Liver Disease; OLT, orthotopic liver transplantation; TIPS, transjugular intrahepatic portosystemic shunt; UCSF, University of California San Francisco.

**Table 2:** Timeline of hepatocellular carcinoma recurrence in Patient 2.

Date	Clinical Event	Findings and Management
February 2022	Liver transplantation	Deceased-donor orthotopic liver transplantation was performed.
October 2023	First detection of suspicious pulmonary nodules	Small bilateral pulmonary nodules were considered suspicious for metastatic disease. Cytologic examination of bronchoalveolar lavage fluid showed no malignant cells.
April 2025	Follow-up imaging	Enlargement of the pulmonary nodules was observed, including a right upper-lobe lesion measuring 22 × 14 mm. Additional abdominal lesions were also reported.
June 2025	Positron-emission tomography	PET demonstrated low fluorodeoxyglucose uptake in the pulmonary lesions.
November 2025	Pulmonary resection	Atypical resections of the right pulmonary lesions were performed because of persistent radiologic suspicion and interval progression.
November 2025	Histological confirmation	Histologic and immunohistochemical examinations confirmed metastatic hepatocellular carcinoma.
January 2026	Initiation of systemic therapy	Treatment with lenvatinib was initiated.
April 2026	Subsequent follow-up imaging	CT demonstrated enlargement of a left lower-lobe metastatic nodule to 9 mm and progression of multiple peritoneal lesions.

Histological confirmation was delayed because the initial cytologic examination of bronchoalveolar lavage fluid was negative for malignant cells and subsequent PET demonstrated low fluorodeoxyglucose uptake; tissue diagnosis was ultimately obtained after interval radiologic progression and persistent suspicion of metastatic disease. Abbreviations: CT, computed tomography; PET, positron-emission tomography.

**Table 3:** Perioperative factor VIII dosing, coagulation monitoring, and blood component use.

Item	Patient 1	Patient 2
FVIII concentrate	Efmoroctocog alfa (extended-half-life rFVIII Fc fusion protein)	Efmoroctocog alfa (extended-half-life rFVIII Fc fusion protein)
Target perioperative FVIII activity	Approximately 100 IU/dL	Approximately 100 IU/dL
Preoperative dose	4000 IU (51.3 IU/kg), 45 min before skin incision	4000 IU (55.6 IU/kg), 30 min before skin incision
Additional intraoperative dose	2000 IU (25.6 IU/kg), approximately 1 h after reperfusion	2000 IU (27.8 IU/kg), approximately 45 min after reperfusion
Postoperative days 1–3	2000 IU every 12 h	2000 IU every 12 h
Once-daily tapering	2000 IU on days 4–6; 1500 IU on days 7–10; 1000 IU on days 11–13	2000 IU on days 4–7; 1500 IU on days 8–11; 1000 IU on days 12–14
Total FVIII administered	33,000 IU	35,000 IU
Inhibitor history/pretransplant assay	No known history; formal assay results unavailable	No known history; formal assay results unavailable
Intraoperative monitoring	Hb, platelets, PT, INR, aPTT, fibrinogen, FVIII activity (before surgery, anhepatic phase, after reperfusion, end of surgery); TEG (baseline, anhepatic phase, after reperfusion)	Same as Patient 1
Fresh-frozen plasma	4 units	3 units
Platelets	1 apheresis unit	Not transfused
Packed red blood cells	2 units	2 units
Other hemostatic agents	None	None
Rescue FVIII doses; bleeding or thrombosis	None; none	None; none

Other hemostatic agents not used: fibrinogen concentrate, cryoprecipitate, tranexamic acid, prothrombin complex concentrate, and recombinant activated factor VII. Abbreviations: aPTT, activated partial thromboplastin time; FVIII, factor VIII; Hb, hemoglobin; INR, international normalized ratio; PT, prothrombin time; rFVIII, recombinant factor VIII; TEG, thromboelastography.

## Data Availability

All relevant de-identified clinical information is included in the manuscript and Table 1, Table 2 and Table 3. Additional patient-level data are not publicly available because of confidentiality requirements but may be available from the corresponding author where legally and institutionally permissible.
